# Eye Movements During RAN as an Operationalization of the RAN-Reading “Microcosm”

**DOI:** 10.3389/fnhum.2020.00067

**Published:** 2020-03-03

**Authors:** Jessica Lee Peters, Edith Laura Bavin, Sheila Gillard Crewther

**Affiliations:** ^1^Department of Psychology and Counselling, La Trobe University, Melbourne, VIC, Australia; ^2^Intergenerational Health, Murdoch Childrens Research Institute, Melbourne, VIC, Australia

**Keywords:** eye movements, rapid naming, reading, dyslexia, young children, ocular motor patterns, microcosm

## Abstract

Rapid Automatized Naming (RAN) is a strong predictor of reading aloud, though there is little agreement on what underpins RAN or how it relates to reading. Some theorize phonological skills, while others suggest that RAN reflects the “microcosm” of cognitive and attentional processes also required for reading, with more recent research using eye movements in an attempt to study this relationship. In the current study, we aimed to extend previous investigations to identify whether the temporal patterns of eye movements predict RAN and can, therefore, be established as a method to study the cognitive processes underlying RAN that could then be utilized to elucidate the relationship of RAN to reading. A Gazepoint eye tracker was used to record the eye movements of 93 learner readers aged 5–8 years (*M* age = 7.00) while performing a custom computerized alphabetic RAN task. Text reading accuracy, comprehension and rate; nonverbal intelligence; and phonological awareness abilities were also assessed. Regression analyses showed that, independently of phonological awareness, eye movements [Fixation Count (FC) and Fixation Duration (FD)] measured during RAN tasks were highly reflective of children’s rapid naming performance (92.8%). Both mean FC and mean FD during RAN tasks also predicted text reading accuracy (36.3%), comprehension (31.6%), and rate (36.2%) scores, and in predicting these text reading skills there was a high level of shared variance with RAN performance. In a sub-sample of participants, longer average FDs and counts independently discriminated children with reading difficulties (*n* = 18; aged 7–9) from neurotypical children matched for age (*n* = 18), but not from younger neurotypical children matched for reading level (*n* = 18; aged 5–6). Together, these results suggest that the analysis of eye movements recorded during RAN allows for the operationalization of many of the spatially and temporally-bound cognitive and attentional processes that underpin the RAN, and a step towards elucidating its relationship to reading.

## Introduction

Rapid Automatic Naming (RAN) is commonly used to measure the ability to rapidly, accurately, and sequentially name a series of repetitive and familiar visual stimuli (i.e., pictures, colors, letters or digits; Denckla and Rudel, 1974). RAN tasks are also known to successfully differentiate those individuals with and those without diagnosed reading difficulties (i.e., specific learning disorder in reading, developmental dyslexia; Denckla and Rudel, 1974). However, until the last few years, there has been little consensus about how RAN relates to reading (for a review, see Kirby et al., 2010), Indeed, early interpretation of the RAN-reading relationship was associated with an impaired ability to make adequate visual to verbal conversions (letter-sound conversions) during RAN and reading, thus limiting the automaticity of access to the phonological representation and impairing task performance (Torgesen et al., 1997; Clarke et al., 2005; Vukovic and Siegel, 2006; Savage et al., 2007; Ziegler et al., 2010). A second common interpretation has been that RAN reflects more a microcosm of the multiple cognitive and attentional skills required for reading (Denckla, 1988), which must take place in the context of sequentially organized eye movements. Recent studies have attempted to elucidate these hypotheses by investigating the individual differences in eye movements as a means to identify which cognitive processes may contribute to RAN (Jones et al., 2008, 2010, 2013; Pan et al., 2013; Yan et al., 2013; Al Dahhan et al., 2014, 2017). However, whether eye movements during RAN are reflective of, and hence predictive of overall RAN performance has not yet been fully elucidated. If indeed the eye movement characteristics are not strong predictors, then using gaze technology to study cognitive processing during RAN would be theoretically uninformative. Thus, the current study aimed to first establish whether RAN performance is dependent on the mean duration and number of fixations needed to name each RAN stimuli, which should lead to confirmation that eye movements can be used to operationalize and measure the time needed to accomplish the cognitive and attentional processes that underpin RAN. Such understanding of the time constraints needed for successful familiar object recognition and verbalization during RAN will add information to how RAN is related to reading fluency and why those with reading difficulties often perform poorly on RAN tasks.

Much of the earliest work relating to eye movements and cognitive demands in tasks related to reading was pioneered by Rayner (1998). For example, fixations are longer and saccade sizes are shorter during oral reading as compared to silent reading (Rayner, 1998; Kim et al., 2019), while saccades, regressions, and Fixation Durations (FDs) increase with greater visual/orthographic similarity during RAN (Al Dahhan et al., 2017). Recent research has also shown that individual differences in temporally-based *fixation durations* are indicative of duration of attentional engagement related to speed of visual, symbolic, and orthographic processing and potentially include time to access the lexicon and verbalize the stimuli (Eckstein et al., 2017; Kim et al., 2019). *Mean fixation counts* per stimuli have been suggested to measure spatial distribution of attention indicative of the amount of visual information processed in each fixation (Goldberg and Kotval, 1999; Holland and Komogortsev, 2011; Rayner et al., 2013), while *saccade duration*, a measure dependent on speed of activation and time to move to the spatial location of the next stimuli to be attended (Baloh et al., 1975), may provide insights into the cognitive processes that take place between fixations during RAN and reading. Neural networks associated with eye movement control and attention are similarly activated in both RAN and reading (i.e., the “reading network”; Misra et al., 2004), leading to the suggestion that RAN could be considered as a surrogate measure of the efficiency of this “reading network” (Al Dahhan et al., 2016), and that eye movements could provide insight into the cognitive and attentional processes important to both RAN and reading.

Furthermore, children and adults diagnosed with a reading disorder are consistently reported to display less efficient patterns of eye movements during RAN and reading tasks, i.e., smaller perceptual spans, longer and more fixations per word, shorter saccades, and more regressions when compared with age-matched typical readers (Rayner, 1986; Ashby and Rayner, 2004; Jones et al., 2008; Logan, 2009; Hawelka et al., 2010; Jones et al., 2010; Moll and Jones, 2013; Pan et al., 2013; Yan et al., 2013; Al Dahhan et al., 2014, 2017; Kuperman et al., 2016; Henry et al., 2018). Such differences in gaze patterns have been interpreted to reflect that those with reading difficulties require more attentional resources and time to attend and engage cognitive mechanisms in order to process information during fixations than normal age-matched readers. However, while many now argue that eye movements can be used to investigate the cognitive processes involved in RAN and reading (Al Dahhan et al., 2016; Eckstein et al., 2017; Kim et al., 2019), there is only limited research specifically exploring how well RAN eye movements predict RAN performance or reading outcomes. Establishing this would aid in confirming that using eye movements to study cognitive processing during RAN is useful in understanding the RAN-reading relationship.

Currently, we are only aware of two studies by Al Dahhan et al. (2014), that have reported on the extent to which eye movements recorded during RAN may predict single word reading and RAN performance. Al Dahhan et al. (2014) found that FD and count recorded during RAN significantly predicted reading in adults, while Al Dahhan et al. (2016) demonstrated that FD during rapid naming, predicted reading and RAN performance in children (aged 6–7 and 9–10) and concluded that RAN and reading are related *via* eye movements which reflect the time required to extract and process stimulus information. While the aims of both articles were to investigate the predominant theories of RAN *via* visual and phonological manipulation of RAN tasks, rather than investigate the role of eye movements, these previous results provide impetus for further investigations to establish such a role for text reading (Araújo et al., 2015; Papadopoulos et al., 2016) rather than single-word reading as used by Al Dahhan et al. (2014, 2017). The close relationship known between RAN and oral text reading is presumably because both skills draw on similar cognitive processes of visual stimulus identification and rapid sequential processing (Araújo et al., 2015; Papadopoulos et al., 2016)—skills less required for single word reading lists. This would suggest that RAN-based eye movements are likely to be more predictive of text reading skills as compared with single-word reading, necessitating the current study.

Thus, in the current study, we aimed to extend upon the works of Al Dahhan et al. (2014, 2017) to further clarify two aspects regarding the role of eye movements during RAN as a way to measure the RAN-reading cognitive “microcosm.” A serial alphabetic RAN task was chosen because this type of RAN task most strongly predicts single reading across development (van den Bos et al., 2002). The first aim was the role of eye movements during a serial alphabetic RAN task and their relationship to RAN and oral text reading performance. We investigated this in a broad sample of primary school-aged learner readers by:

1. examining how well eye movements recorded during RAN predict RAN performance;
2. examining the extent with which eye movements and phonological awareness separately predicted RAN, to demonstrate whether RAN is more reflective of phonological processes or the cognitive “microcosm” eye movements are believed to reflect;
3. determining the unique contribution of RAN-based eye movements in predicting text reading accuracy, rate and comprehension performances and;
4. identifying the shared contributions between RAN and RAN-based eye movements as overlapping predictors of text reading performances, in order to further establish that eye movements can be utilized as proxy measures of RAN and as a means of identifying the microcosm of cognitive processes that underlie RAN and the RAN-reading relationship.

The second focus was on discriminating reading difficulties using eye movements, and in this aspect of the research we aimed to:

5. identify whether eye movements during RAN discriminate children with reading difficulties from chronological- and reading-age matched normal readers, which would further indicate that eye movement are useful measures of the cognitive processing underpinning reading development.

Based on the findings of previous research, we hypothesized that eye movement patterns during RAN would prove highly reflective of RAN performance, so would strongly predict RAN performance, and to a greater extent than phonological awareness. It was also hypothesized that eye movements during RAN would significantly predict text reading performances (accuracy, comprehension, and rate) more strongly than for the single words as used by Al Dahhan et al. (2014, 2017) and that the predictive contribution of RAN eye movements on text reading would largely overlap with the contribution provided by RAN performance. It was also hypothesized that eye movements would successfully differentiate children with reading difficulties from chronological-, and reading-age matched normal readers, providing further evidence that individual differences in eye movements are related to both RAN performance and the cognitive processes involved.

## Materials and Methods

### Participants

For the first part of the study, ninety-three primary school children (52 male) aged 5 years to 9 years 2 months (mean age = 7.00, SD = 0.99), from Prep (i.e., the first year of formal schooling; *n* = 32), Grade 1 (*n* = 35), and Grade 2 (*n* = 26) participated in the study. Participants were tested towards the end of the school year to ensure that children in Prep had received close to 1 year of formal instruction of word and sentence reading. Participants were recruited from mainstream primary schools and an extracurricular program for children with diagnosed specific reading disorders to ensure the sample was representative of the full reading spectrum. All participants had normal intelligence (Standard score ≥85 for age), normal or corrected-to-normal vision and hearing, and English as their primary language. The sample included 23 participants diagnosed with specific reading difficulties (i.e., specific learning disorder in reading and/or developmental dyslexia), which was confirmed *via* standardized assessment (Reading performance >1.5 SD below age norms; O’Brien et al., 2012; American Psychiatric Association, 2013). Children with known medical and neurodevelopmental disorders other than developmental dyslexia or specific reading disorder were excluded (see DSM-5; American Psychiatric Association, 2013). Table 1 provides descriptive statistics for all measures of interest.

**Table 1:** Participants means and standard deviations for reading related measures and eye movements.

	*M*	*SD*	Min.	Max.
RCPM	115.96	9.45	91.00	125.00
Phonological awareness	104.16	15.27	70.00	145.00
RAN (raw score)	72.77	20.79	19.00	113.00
Reading accuracy	99.46	18.10	65.00	135.00
Reading comprehension	94.99	16.55	65.00	131.00
Reading rate	103.75	19.70	65.00	145.00
Fixation duration (ms)	442.37	71.50	270.22	510.00
Fixation count	1.71	0.35	1.13	2.52
Saccade duration (ms)	54.64	21.49	20.03	100.00

*Note. Reading, phonological awareness, and RCPM means and SD’s represent standard scores. ms, milliseconds; RCPM, Ravens Color Progressive Matrices*.

For the second part of the study, a sub-sample of the recruited participants (*n* = 54) were further investigated in order to compare the eye movement patterns of those with and without reading difficulties. Children with reading difficulties (RD; aged 7–9; *n* = 18) were compared to chronological-age-matched controls (CA; aged 7–9; *n* = 18) and reading-age-matched controls (RA; aged 5–6; *n* = 18). RD children were one-to-one matched with both control counterparts (CA and RA) on age-standardized nonverbal intelligence (*z* = ± 0.8), with CA children within 1 year of age, and with RA children within 1 year of reading age. An *a priori* power analysis indicated that this sample size was sufficient to detect a large effect size with 95% power.

### Procedure

The research was carried out in accordance with ethics approval granted by the La Trobe University Faculty Human Ethics Committee and the Victorian State Department of Education. Parents of participants were required to provide written informed consent for their child to engage in the study. All children voluntarily participated. Testing occurred in a small quiet room, over approximately two 30-min sessions at the participants’ school or program, with tasks administered in randomized order.

### Materials

#### Nonverbal Intellect

The Raven’s Colored Progressive Matrices (RCPM) test was used to assess nonverbal reasoning (Raven et al., 1998). The RCPM contains three series of 12 matrices of increasing complexity. Standard scores were calculated based on chronological age using normative data provided in Cotton et al. (2005). The RCPM is standardized in a range of countries including Australia and is considered appropriate for children of ages 5–11 years and for children with reading difficulties (Cotton et al., 2005). The Raven’s exhibits good test-retest reliability (*r* = 0.80; Raven et al., 1998) and high internal consistency (*α* = 0.89), with minimal variation across age (Cotton et al., 2005).

#### Reading Ability

Reading was measured with the Neale Analysis of Reading Ability—Third Edition, which is a standardized test of reading ability for children in Grades Prep to 6, commonly used in Australian school settings (Neale, 1999). The test measures reading accuracy, comprehension, and rate during prose oral reading *via* a series of up to six passages of increasing difficulty with accompanying questions. Children were first required to complete a practice passage, and all children were able to participate in the test. Grade-based standard scores for reading accuracy, comprehension and rate were calculated from the raw scores based on the manuals’ normative data. Internal consistency results vary by age, with α ranging from 0.86 to 0.92 for comprehension, 0.91–0.97 for accuracy and 0.71–0.94 for rate (Neale, 1999). The overall measure has high content validity and face validity for the construct of reading aloud and is effective in discriminating between ages and differing reading abilities, including poor reading and dyslexia.

#### Phonological Awareness

Phonological knowledge was assessed using the Elision subtest, a sound deletion task, from the Comprehensive Test of Phonological Processing (CTOPP; Wagner et al., 1999). The age-based standard score was used as the measure of phonological awareness. It demonstrates good internal consistency (α =.91), test-retest (*α* = 0.82), and inter-rater reliability (*r* = 0.96), and has high concurrent validity with other tests of phonological processing (Wagner et al., 1999).

#### Rapid Automatized Naming

**Figure 1:**
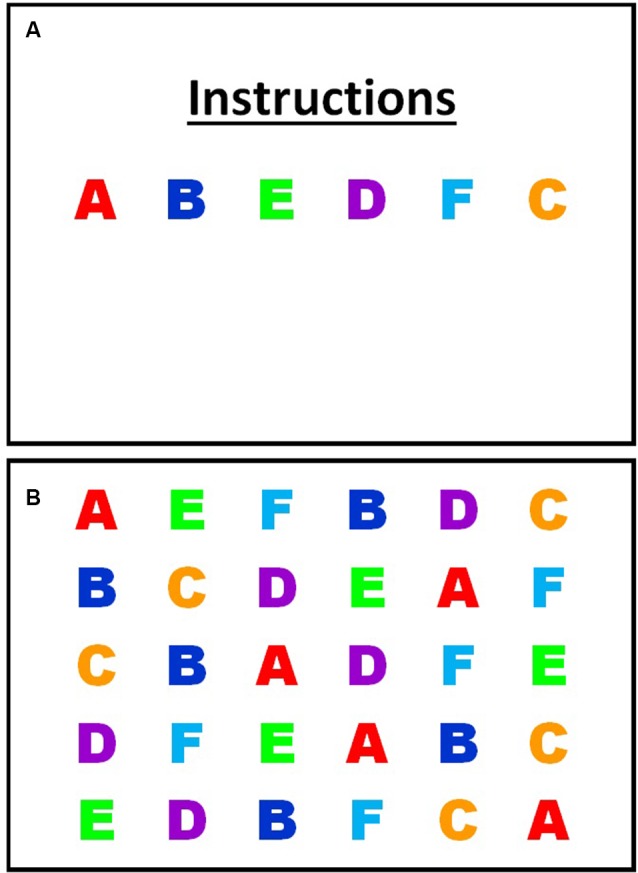
Rapid Automatic Naming (RAN) practice trial **(A)**; RAN timed trial **(B)**.

The custom serial letter RAN task employed here consisted of 30 items of six randomly repeated letters (see Figure 1). RAN performance was recorded as a number of stimuli named in 60 s, rather than time to complete, as used in most other RAN tasks. A performance indicator of RAN that controlled for the time was chosen as most of the eye movement variables included were time-based, while the 60 s time duration was selected to ensure that the averaged eye movement variables were representative. RAN tasks require stimuli to be named in a quick, automatic manner, so the uppercase letters A through F were chosen as stimuli because uppercase letters and letters from the beginning of the alphabet are learned earliest (McBride-Chang, 1999; Justice et al., 2006), so would be automatized earliest. Consistent with other alphabetic RAN tasks, each of the chosen stimuli were single-syllable. The task was presented as a single frame on a computer screen, and participants sat at a viewing distance of 60 cm. The visual angle of each letter was 2 × 2°. Participants were first provided a practice trial showing all six-letter stimuli to ensure they could name each letter without error and to familiarize them with the requirements of the task. Participants unable to accurately complete the practice trial were discontinued from the task. Participants were instructed to name aloud the stimuli as fast and as accurately as possible, from left to right, top to bottom, and repeating through the 30 stimuli as many times as possible, and self-correcting any errors, until the display disappeared (60 s). The total number of stimuli named was recorded manually. Eye-tracking data was then analyzed for the duration (60 s) of the task. Eye movements during naming errors were not removed from the data.

#### Eye Movement Patterns

Eye movements were recorded binocularly during the RAN task using a Gazepoint GP3 screen mounted infrared camera (60 Hz sampling rate; Gazepoint^1^). The GP3 tracks vertical and horizontal eye positions with an average gaze position accuracy of 0.5°. Participants were positioned 60 cm from the screen with their head placed in a chin and forehead rest to reduce movement. Before beginning the task, each participant underwent a 9-point eye movement calibration procedure. FD was calculated as the average (mean) temporal length the fixations performed during the 60 s RAN task. Saccade Duration (ScD) was calculated as the average (mean) duration (in milliseconds) of saccades performed during the 60 s RAN task. This variable was chosen as it provides a summary measure of saccadic function (i.e., reflective of the speed of activation and time required to move the eyes to the next fixation location) that permitted investigation of eye movements more broadly while minimizing the number of variables included in analyses. FC was defined at the average number of fixations required per stimuli named and was calculated by dividing the total number of fixations made by the total number of letters named during the RAN task. As the RAN task used a fixed time limit rather than number of stimuli, the FC variable controls for individual participant RAN score differences (i.e., differences in the number of letters named), and so is akin to FC measures used in experiments presenting a fixed number of stimuli.

## Data Screening and Analysis

Data screening identified a total of 12 outliers across the eye movement measures (FD = 3; ScD = 6; FC = 3) that were just outside the normal distribution (i.e., ~4% of the eye movement data). To reduce this influence on parametric statistical analyses, outliers were pulled back to the next most extreme value within the normal distribution (Tabachnick and Fidell, 2013). Further assumption testing revealed no other violations.

Correlation analyses were conducted to determine which, if any, eye movement measures related to RAN and to inform which to include in the regressions. High correlations between the eye movements variables and RAN performance were found, suggestive of non-independence between the variables. This was not unexpected as the eye movements were recorded during the RAN task. Although multicollinearity between predictor variables is typically addressed by removal of one of those variables from the regression model, this was not performed in the current study given that multicollinearity has been shown to not reduce the reliability or predictive power of the regression model, rather only reducing the likelihood that individual predictors will be statistically significant (Allen, 2004). Therefore, a series of hierarchical multiple regressions were conducted to investigate what contribution eye movement patterns may make to RAN and to text reading ability (i.e., accuracy, comprehension, and rate) in young readers. The regression analysis for RAN included phonological awareness and the chosen eye movement variables to allow direct comparison of their contributions, and to identify whether RAN is more reflective of phonological processes or the cognitive “microcosm” eye movements are believed to reflect. In each regression model for text reading (accuracy, rate, comprehension), the aim was to determine the unique contribution that eye movements provide to the reading skills, as well as the overlap in the contribution of eye movements and RAN, to reading. Other variables that are known to be important to reading, such as phonological awareness, were not included in the reading regressions as this has been previously investigated (see Al Dahhan et al., 2014). Eye movements were entered at step 1 to determine specifically what unique contribution they made independent of the broader RAN performance variable. RAN performances were then entered at step 2 to determine what further contribution RAN made to the reading models and how much variance contributed by eye movements and RAN was shared.

For the second part of our research, reading subgroups were compared using one-way analysis of variance (ANOVA’s) to ascertain whether eye movements could differentiate between a group of children with reading difficulties, a matched group of chronological-aged normal readers, and a group matched on reading-age.

## Results

### The Relation of Eye Movements to Rapid Naming Performance and Reading

Pearson correlational results show that FD and FC correlated significantly with nonverbal intelligence, RAN, phonological awareness and all reading measures (see Table 2). Saccade duration did not correlate with these measures.

**Table 2:** Correlations between rapid naming, reading, phonological awareness, nonverbal intelligence, and eye movement patterns.

	2.	3.	4.	5.	6.	7.	8.	9.
1. RCPM	0.311**	0.448**	0.321**	0.335**	0.235*	−0.285**	−0.250*	−0.001
2. Rapid naming	-	0.377**	0.642**	0.600**	0.613**	−0.682**	−0.874**	−0.102
3. Phon. awareness	-	-	0.587**	0.497**	0.428**	−0.307**	−0.286**	−0.065
4. Reading accuracy	-	-	-	0.831**	0.823**	−0.437**	−0.540**	−0.065
5. Reading comp.	-	-	-	-	0.729**	−0.423**	−0.494**	−0.101
6. Reading rate	-	-	-	-	-	−0.502**	−0.486**	−0.019
7. Fixation duration	-	-	-	-	-	-	0.347**	−0.272**
8. Fixation count	-	-	-	-	-	-	-	0.098
9. Saccade duration	-	-	-	-	-	-	-	-

Note. *p ≤ 0.05, **p ≤ 0.01. RCPM, Ravens Color Progressive Matrices; Phon. Awareness, phonological awareness.

### Predictors of Rapid Naming

The independent eye movement variables, FC and FD, were chosen for the hierarchical multiple regression for RAN performance based on the significant correlations shown in Table 2. Phonological awareness was included based on past theoretical considerations of its importance to RAN. Therefore, phonological awareness, FD and FC were entered together as predictors of letter RAN performance.

The results in Table 3 show that only the two eye movement measures (FD and FC), and not phonological awareness, were significant predictors of RAN performance, together explaining 92.8% of the variance in the regression model. These results indicate that eye movements—namely shorter and fewer fixations made for each stimulus named—are highly predictive of the rate of rapid naming performance in young readers, with more efficient eye movements relating to better performance outcomes and so should be considered as discrete substitute measures of RAN.

**Table 3:** Predictive contributions of phonological awareness and eye movement patterns on alphabetic rapid naming performance.

Alphabetic rapid naming performance	*β*	*r*	*sr*
Phonological awareness	0.04	0.38	−0.04
Fixation duration	−0.42**	−0.68	−0.38
Fixation count	−0.72**	−0.87	−0.66
Total *R*^2^ = 0.928, *F*_(3,84)_ = 362.293, *p* < 0.001			

Note. **p ≤ 0.001; according to Cohen’s guidelines, r ≥ 0.10, r ≥ 0.30, and r ≥ 0.50, represent small, medium, and large effect sizes, respectively (Cohen, 1988).

### Predictors of Reading Ability

Hierarchical multiple regressions were conducted for each text reading skill, despite the dependent variables (reading accuracy, comprehension, and rate) being highly correlated (see Table 2), because the contributions of RAN-based eye movements to each of the three aspects of text reading is not fully known. For each analysis, FD and FC were entered as predictors at Step 1 to first establish the contribution of these discrete functions given their overlap with RAN as shown in the previous analyses, with RAN performance then entered at Step 2 to determine how much more variance it may contribute to the text reading analyses. Assumption testing revealed no violations. Table 4 presents the results of each reading regression (reading accuracy, comprehension, and rate) respectively.

**Table 4:** Predictive contributions of eye movement patterns and RAN on reading accuracy, reading comprehension, and reading rate.

		Reading accuracy			Reading comprehension			Reading rate		
		β	*r*	*sr*	β	*r*	*sr*	β	*r*	*sr*
Step 1:	Fixation duration	−0.28*	−0.44	−0.27	−0.29*	−0.42	−0.19	−0.38**	−0.50	−0.36
	Fixation count	−0.44**	−0.54	−0.41	−0.40**	−0.49	−0.30	−0.35**	−0.49	−0.33
		*R*^2^ = 0.363**, *F* change (2,89) = 25.34			*R*^2^ = 0.316**, *F* change (2,89) = 20.59			*R*^2^ = 0.362**, *F* change (2,859) = 24.12		
Step 2:	Fixation duration	0.08	−0.44	0.04	0.07	−0.42	0.03	−0.12	−0.50	−0.06
	Fixation count	0.17	−0.54	0.06	0.20	−0.49	0.07	0.09	−0.49	0.03
	Rapid letter Naming	0.84*	0.64	0.23	0.82*	0.60	0.22	0.61	0.61	0.17
		Change *R*^2^ = 0.052*, *F* change (1,88) = 7.87			Change *R*^2^ = 0.049*, *F* change (1,88) = 6.79			Change *R*^2^ = 0.028, *F* change (1,88) = 3.79		
		Total *R*^2^ = 0.415**, *F*_(3,88)_ = 20.82			Total *R*^2^ = 0.365**, *F*_(3,88)_ = 16.88			Total *R*^2^ = 0.390**, *F*_(3,88)_ = 17.87		

Note. *p ≤ 0.05, **p ≤ 0.001; according to Cohen’s guidelines, r ≥ 0.10, r ≥ 0.30, and r ≥ 0.50, represent small, medium, and large effect sizes, respectively (Cohen, 1988).

The total variance explained by the reading accuracy regression model was 41.5%. FD and FC explained 36.3% of the variance at step 1, with RAN then explaining an additional 5.2% at step 2. The total reading comprehension analysis explained 36.5% of the variance. FD and FC together explained 31.6% of the variance at step 1, and when entered at step 2, RAN performance explained an extra 4.9% of the variance. The total reading rate regression model explained 39.0% of the variance. At step 1, the two eye movement measures explained 36.2% of the variance, while RAN performance explained an extra 2.8% of the variance in step 2, although this was not a significant contribution.

However, when independent variables were considered separately the significance of eye movement measures no longer remained in any of the three final regression models. This is most likely due to the high level of overlap between RAN and the eye movement measures, as shown in the previous correlation and RAN regression analyses. In the final regression analyses for text reading, RAN was the only significant and individual predictor for Reading Accuracy and Comprehension, while no variable remained a significant unique predictor for Reading Rate.

### Reading and Age Comparisons Between Those With and Without Reading Difficulties

Results of initial group comparisons confirmed that the three groups were appropriately comparable. Preliminary analyses revealed no assumption violations. Raw scores for reading were used to facilitate comparisons during analyses; however standard scores and age-equivalents for reading have been provided in Table 5 to aid meaningful interpretation. Groups did not differ on age-standardized nonverbal intelligence (i.e., Raven’s; *F*_(2,50)_ = 0.42, *p* = 0.659, *d* = 0.25). The RD and CA groups did not differ in chronological age (*F*_(2,50)_ = 44.05, *p* > 0.001, *d* = 2.65; Tukey HSD *post hoc* comparisons showed only the RA group differed significantly from the RD and CA groups), while the RD and RA groups did not differ on reading age or phonological awareness, with only the CA group performing significantly better than the RD and RA groups (Reading accuracy, *F*_(2,50)_ = 30.35, *p* < 0.001, *d* = 2.20; comprehension, *F*_(2,50)_ = 28.23, *p* < 0.001; *d* = 2.24; rate, *F*_(2,50)_ = 21.66, *p* < 0.001, *d* = 2.01), and phonological awareness, *F*_(2,50)_ = 6.45, *p* = 0.003, *d* = 1.06). Statistically significant differences between groups for RAN performance were also found (*F*_(2,50)_ = 8.08, *p* = 0.001, *d* = 1.14), with the CA group performing better than the RD group.

**Table 5:** Participants means and standard deviations for age, nonverbal intelligence, and reading related measures.

	Reading disorder group (*n* = 18)		Chronological-age-matched control group (*n* = 18)		Reading-age-matched control group (*n* = 18)	
	*M*	*SD*	*M*	*SD*	*M*	*SD*
Age in years	7.71	0.78	7.65	0.63	5.91	0.50
RCPM SS	107.72	10.13	110.52	6.88	109.11	9.71
RAN (raw score)	59.89	17.12	84.47	19.88	70.72	17.28
Phon. awareness SS	87.35	7.09	107.00	14.24	102.35	11.06
Phon. awareness age equiv	6.36	0.70	9.18	2.80	6.28	0.87
Reading accuracy SS	75.00	8.08	110.35	12.16	102.44	6.50
Reading accuracy age equiv	6.21	0.41	8.57	2.12	6.37	0.35
Reading comprehension SS	77.55	11.71	101.00	13.63	94.89	11.81
Reading comprehension age equiv	6.30	0.54	7.62	0.97	6.25	0.37
Reading rate SS	80.83	13.66	118.07	15.15	104.88	12.45
Reading rate age-equiv	6.63	1.13	10.00	2.38	6.72	0.87

*Note. SS, Standard score; RCPM, Ravens Color Progressive Matrices; Phon. Awareness, phonological awareness; Age Equiv, age equivalent score*.

### Comparisons of Eye Movements During Rapid Naming in Children With and Without Reading Difficulties

**Figure 2:**
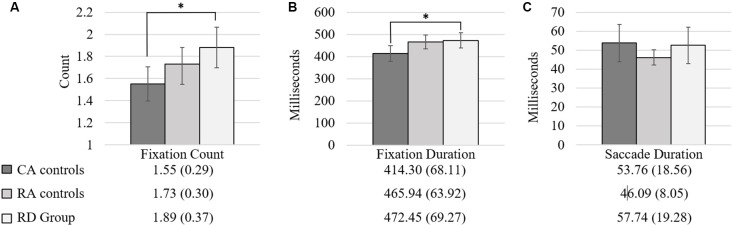
Group differences for fixation count (FC; **A**); fixation duration (FD; Milliseconds; **B**); and saccade duration (Milliseconds; **C**). *Note*. *p ≤ 0.05; 95% Confidence interval error bars.

One-way ANOVA comparisons of the eye movement patterns of children with reading difficulties, chronological-age matched controls and reading-age matched controls demonstrated statistically significant differences between groups for FD (*F*_(2,50)_ = 3.90, *p* = 0.027, *d* = 0.80) and FC (*F*_(2,50)_ = 4.66, *p* = 0.014, *d* = 0.87), with large effect sizes found. There were no differences between groups for ScD (*F*_(2,50)_ = 2.45, *p* = 0.097, *d* = 0.63). *Post hoc* comparisons using the Tukey HSD test indicated that the CA group differed significantly from the RD group in FD (414.30 vs. 472.45 ms) and FC (1.55 vs. 1.89 fixations), with chronological-age-matched controls making more fixations on the RAN task with shorter average duration of fixations and fewer fixations per stimulus than those with reading difficulties. Neither group differed significantly from the reading-age-matched controls in FD (465.94 ms) or FC (1.73 fixations). Figure 2 depicts the performance of each reading group for the Fixation Count (FC; Figure 2A), Fixation Duration (FD; Figure 2B), and Saccade Duration (ScD; Figure 2C).

## Discussion

The current study examined eye movement patterns during rapid naming in young children to better elucidate the extent to which the temporal constraints in eye movements and attention shifting predict and can, therefore, be considered reflective of RAN performance. We are assuming that if eye movements during RAN explain significant variance in RAN performance, this should establish that eye movements can be used to operationalize and temporally sequence the microcosm of attentional and higher cognitive processes required for successful object recognition and verbalization as in RAN. Such knowledge also facilitates understanding of the relationship between RAN and oral text reading. The results provide evidence in support of the notion that RAN and text reading ability (accuracy, rate and comprehension) can be significantly predicted by the efficiency of eye movement behavior during RAN in 5-8-year-old children and that these eye movements also successfully differentiate age-matched children with and without reading difficulties. Moreover, our findings indicate that the average FD and FC per RAN item named is highly predictive of RAN, and that these eye movements and RAN show a strong overlap in their predictive contributions to text reading, suggesting that eye movements recorded during RAN reflect much of the cognitive processing required by both RAN and reading. Our interpretation of these measures is based on research (Eckstein et al., 2017; Kim et al., 2019) demonstrating that individual differences in temporally-based *fixation durations* are indicative of the duration of attentional engagement related to the speed of visual, symbolic, and orthographic processing. By comparison, average *FCs* per stimuli provide a measure of the spatial distribution of attention indicative of the amount of visual information processed in each fixation.

### What Predicts Rapid Naming?

Duration and count of fixations (FD and FC) were recorded during RAN and were found to contribute significantly to RAN performance (92.3%), raising the question of variable independence. Eye movement variables have been interpreted as highly reflective of overall RAN performance rather than as independent, individual predictors. Since our results indicated that eye movements did not entirely account for RAN performance, additional factors must contribute to RAN performance. Our results are consistent with those reported by Al Dahhan et al. (2016), who found that FD, saccade count and number of regressions accounted for 83% of the variance in rapid naming. Indeed our findings also reiterate meta-analytical evidence (Swanson et al., 2003) showing that while phonological awareness and RAN correlate, they load to separate factors of reading indicative of an inadequate explanation for rapid naming ability and suggestive that FD times are not solely mediated by the time needed for phonological activation and retrieval at each fixation. Other evidence against a phonological interpretation comes from Compton (2003) who showed that increasing the visual (orthographic) similarity of the letters within a RAN task negatively affected performance to a much greater extent than increasing phonological similarity. Furthermore, Georgiou et al. (2013) showed that while rapid discrete naming of stimuli (presented one-at-a-time) has similar phonological processing requirements to rapid serial naming of multiple stimuli (presented in an array), it is less well correlated to reading. The relationship between RAN and reading also increased considerably when the “naming” aspect of RAN was accounted for by controlling the effect of discrete RAN on serial RAN performance, suggesting that speed of lexical access does not significantly mediate the RAN-reading relationship (Logan et al., 2011). Consistent with this research, our findings show that eye movement patterns, specifically the amount of time needed to acquire information (FD) and how much information is processed at each fixation (FC), are the most important factors in predicting RAN performance, suggesting that it is not phonological skills that are important for RAN ability, but rather the broader cognitive and attentional process (i.e., the “microcosm”) that eye movements incorporate.

### What Predicts Text Reading Skills?

Duration and count of fixations (FD and FC) each made significant contributions to reading accuracy, comprehension, and rate in young readers—together accounting for 36.3%, 31.6%, and 36.2% of the variance respectively. This is higher than the findings of Al Dahhan et al. (2016), who found that eye movement during RAN only accounted for 15% of the variance in word reading skill. We argue that the larger predictive power of RAN-based eye movements in the current study is likely to reflect the use of a text reading measure, rather than word lists, as gaze patterns during RAN would be a closer approximation of the eye movements required in oral text reading.

Entering the eye-movement components into the text reading regressions before RAN, enabled investigation of the unique contributions of eye movements to reading as well as further assessment of the RAN-reading relationship. As expected, once FD and FC had been accounted for, RAN only contributed a further 5.2% of variance to reading accuracy, 4.9% to comprehension and no further significant variance to reading rate. This highlights not only an important overlap of contribution between RAN and the fixation variables to text reading ability but also a small but important contribution of RAN to text reading independent of the variance explained by eye movements. When all predictors were compared once RAN was added to the regression analyses, FD and FC were unsurprisingly no longer significant unique predictors for reading accuracy, comprehension or rate, with RAN becoming the strongest predictor. Thus, the amount of time needed to acquire information (FD) and the number of fixations needed to acquire this information (FC) is closely related to individual differences in reading performance, suggesting that proficiency in fixation behavior can play a role in elucidating much of the relationship between RAN and reading.

### Do Eye Movements Differentiate Children With and Without Reading Difficulties?

Children with reading difficulties were shown to have less proficient fixation characteristics than chronological-age matched controls, with proficiency being measured as the average length of FD and number of fixations (1.89 vs. 1.55 fixations) needed for successful naming of each RAN stimuli. Interestingly, neither of these groups showed eye movement differences when compared to a younger control group (1.73 fixations) who were matched on reading-age to those with reading difficulties. No difference in saccade duration was found between groups. The results are comparable with other eye-tracking studies of RAN (Yan et al., 2013; Al Dahhan et al., 2016). Children with reading difficulties (aged 9–10 years) have been shown to perform significantly worse than age-matched controls for RAN task efficiency errors, FDs, regressive fixations, articulation times, and pause times (Al Dahhan et al., 2016). Similarly, Yan et al. (2013) reported that 10-year-old Chinese children with reading difficulties process less parafoveal information, requiring more attention for local (foveal) processing of individual letters than controls, inevitably inhibiting their ability to anticipate the next character/icon and hence the rate of rapid naming. This would also result in requiring more fixations per stimuli. It appears that those with reading difficulties are generally less efficient than aged-matched normal readers in their eye movement driven temporal processing of information on RAN tasks, despite being familiar with the stimuli; and therefore, apparently requiring more attention and longer fixations for the required cognitive processes.

The less mature eye movement patterns seen in those with reading difficulties may also result from spatial and temporal sequencing deficits associated with impaired magnocellular processing and neural timing (Stein, 2003). It has been suggested that deficient magnocellular neurons are likely to reduce attentional focus, preventing the linked parvocellular neurons from isolating and sequentially processing the relevant information, and resulting in the diffused attentional distribution experienced by those with a reading disorder (Geiger et al., 1994; Facoetti et al., 2000; Lorusso et al., 2004; Lawton, 2007; Laycock and Crewther, 2008; Laycock et al., 2012). This would lead to reduced efficiency in cognitively extracting information during fixations, leading to more fixations, longer fixations and more regressions (Stein, 2003), and highlights the increasing importance of investigating eye movement patterns in both reading research and clinical settings.

### Limitations and Future Directions

The statistical limitation of using a continuous variable (Reading Accuracy on the Neale) to determine group membership in the sub-sample comparison analyses is an important one but was performed with the sound rationale of comparing clinical and neurotypical populations to further inform understanding of reading difficulties (Cohen, 1988). It is also acknowledged that the use of a FC variable partially based on RAN performance (average number of fixations per stimuli named) may pose a statistical limitation influencing the results of the RAN regression. This is of particular importance for samples of more proficient readers who may make a single fixation per stimuli, as this would lead to FC becoming the inverse of the number of RAN stimuli named. However, the current study of emerging readers included children with reading difficulties through to fluent readers, and as such there was a range of variability in FC (i.e., 1.13–2.52 fixations per stimuli; see Table 1) within the sample. It will be important for future research to carefully consider the influence of interdependency of eye movements variables with measures of the task in which they are recorded. What also remains to be further investigated is the influence of the underlying cognitive processes on eye movement patterns and how these processes link to individual eye movement variables during RAN. For instance, there is already some evidence to suggest that the average duration of fixation may reflect the efficiency of visual/orthographic acquisition from the target stimulus (Al-Wabil and Al-Sheaha, 2010; Bellocchi et al., 2013; Al Dahhan et al., 2017). RAN itself also clearly involves well-directed visuo-attention and processing, as well as the speed of orthographic, phonological and semantic identification, and ability to inhibit previously named stimuli, sequentially update, and monitor ensuing information (Executive function; see O’Brien et al., 2012; Al Dahhan et al., 2016). Deficits have been found in those with reading difficulties in each of these aforementioned areas (Ramus et al., 2003; Reid et al., 2007; Menghini et al., 2010). Finally, while the current study does not address the mechanistic link of eye movements and reading, there are already a number of reading intervention studies that target eye movements (see reviews by Bucci, 2019; Peters et al., 2019).

## Conclusion

In summary, the findings of the current study add to the body of evidence supporting the notion that eye movements can be used as surrogate measures to investigate many of the cognitive and attentional processes that underpin the relationship between RAN and reading. While those advocating that RAN and the RAN-reading relationship are predominantly reflective of phonological processes continue to be cited (Wagner et al., 1994; Torgesen et al., 1997; Clarke et al., 2005; Vukovic and Siegel, 2006; Savage et al., 2007; Ziegler et al., 2010), our results add to the literature supporting an alternative explanation (Compton, 2003; Thomson et al., 2006; Powell et al., 2007; Jones et al., 2008; Al Dahhan et al., 2014). Rather, RAN and reading is more likely related by the ability to rapidly process multiple visual stimuli *via* a cognitive “microcosm,” as originally proposed by Denckla (1988). As such behavior can be measured by fixation behavior during RAN, eye movement patterns demonstrated during RAN should provide a way to further elucidate the RAN-reading relationship. Further research into how eye movement measurements can provide real-time insight into the cognitive processes underlying RAN and reading, including mapping cognitive processes to specific eye movements, is the next step in understanding the association between RAN and reading.

## Data Availability Statement

The datasets generated for this study are available on request to the corresponding author.

## Ethics Statement

Ethics approval for the current research was given by La Trobe University Human Ethics Committee and the Victorian Department of Education and Training. Written informed consent to participate in this study was provided by the participants’ legal guardian/next of kin.

## Author Contributions

JP, EB, and SC contributed to the conception and design of the study. JP collected the data, performed the statistical analysis and wrote the first draft of the manuscript. All authors contributed to manuscript revision, and read and approved the submitted version.

## Conflict of Interest

The authors declare that the research was conducted in the absence of any commercial or financial relationships that could be construed as a potential conflict of interest.

## Footnotes

^1^www.gazept.com
